# Cardiovascular Exercise Improves Inhibitory Control in Sedentary Young Adults: A 12-wk Randomized Controlled Trial

**DOI:** 10.1249/MSS.0000000000003751

**Published:** 2025-05-07

**Authors:** MICHAL REMISZEWSKI, GABRIELA RAJTAR, ZUZANNA KOMAREK, TOMASZ PAŁKA, MARCIN MACIEJCZYK, TOMASZ S. LIGEZA

**Affiliations:** 1 Psychophysiology Laboratory, Institute of Psychology, Jagiellonian University, Kraków, POLAND; 2 Institute of Sports Sciences, University of Physical Education, Kraków, POLAND; 3 Department of Physiology and Biochemistry, Faculty of Physical Education and Sport, University of Physical Education, Kraków, POLAND

**Keywords:** COGNITION, EVENT-RELATED POTENTIALS (ERP), FLANKER TASK, N2, PHYSICAL ACTIVITY, P3

## Abstract

**Purpose:**

Physical exercise may enhance cognitive functions, including inhibitory control. Despite increasing evidence, there remains a need for robust evidence on long-term interventions targeting inhibition in healthy, sedentary young adults. We investigated the effects of a 12-wk cardiovascular exercise program on this population’s behavioral and neuroelectric measures of inhibitory control.

**Methods:**

Sedentary young adults were randomized into an experimental group (*n* = 32) or a passive control group (*n* = 30). The experimental group completed a cycling ergometer program consisting of 6 wk of moderate-intensity continuous exercise, followed by 6 wk of moderate- to high-intensity interval exercise. Inhibitory control was assessed at baseline (pretest), after 6 wk (midtest), and after the intervention (posttest) using a modified flanker task with EEG recordings. Assessed outcomes included response time (RT) and event-related potentials (the amplitude and latency of the N2 and P3b components).

**Results:**

The experimental group exhibited a progressive reduction in RT for incongruent trials across all time points (pretest to midtest, midtest to posttest, and pretest to posttest) without compromising accuracy. The control group showed RT reductions only from pretest to posttest, with a decline in overall accuracy. Neuroelectric analyses revealed decreased N2 amplitudes and faster P3b latencies in the experimental group from midtest to posttest during incongruent trials. The control group demonstrated increased N2 amplitudes from pretest to midtest and pretest to posttest during congruent trials.

**Conclusions:**

A 12-wk cardiovascular exercise intervention combining moderate-intensity continuous exercise and moderate- to high-intensity interval exercise enhances both behavioral and neuroelectric indices of inhibitory control in sedentary young adults. These findings highlight the potential of exercise programs as an accessible and effective strategy for improving cognitive health, especially in healthy but sedentary adults.

The growing prevalence of a sedentary lifestyle poses various risks to overall health, such as cardiovascular diseases (1) and certain types of cancer (2). Moreover, a sedentary lifestyle, typical for office work and extended computer use, leads to challenges for cognitive functioning (3). Worldwide, one in four adults does not currently meet the global recommendations for physical activity (PA) set by the World Health Organization (4). Physical exercise, widely recognized as a strategy for enhancing overall health, seems to be an effective solution to the cognitive problems faced by sedentary individuals. Numerous studies have demonstrated that physical exercise can positively impact cognition, including inhibitory control (5), but most of the evidence concerns children, elderly, or active populations. In this study, we examined the effects of long-term physical exercise on inhibitory control in healthy sedentary adults—a group that remains underresearched in this context.

## Inhibitory control

Inhibitory control is a cognitive process that enables individuals to regulate their thoughts, emotions, and actions by suppressing automatic or impulsive responses in favor of goal-directed behaviors (6,7). This ability is particularly crucial during young adulthood, a period marked by increasing independence, complex decision-making, and exposure to novel challenges. Effective inhibitory control supports young adults in navigating social interactions, resisting temptations, managing academic and professional demands, and making adaptive choices under stress (8–11).

Inhibitory control is often measured using conflict resolution tasks, including the Eriksen flanker task (12), Stroop task (13), Go/No-Go task (14), and Simon task (15). The Eriksen flanker task specifically targets the interference aspect of inhibitory control. Interference control involves managing competing or distracting information to maintain focus on a task. It is becoming increasingly important today, enabling us to stay focused and productive despite digital distractions, such as personalized advertisements and social media that compete for attention. People frequently need to filter out irrelevant stimuli—whether it is background noise, competing demands during multitasking, or other distractions—to remain focused and efficient.

During the flanker task, participants are presented with four peripheral (the flankers) and one central (the target) arrow. The flankers always point in the same direction, either left or right. The central target arrow may point in the same direction as the flankers (congruent condition) or in the opposite direction (incongruent condition). Participants’ task is to determine which direction the target is pointing, which requires them to focus on the target while ignoring the distracting flankers. Especially in incongruent conditions, participants must inhibit their reaction to the peripheral arrows and respond to the target, which makes the flanker task an effective tool for measuring inhibitory control (12).

## Markers of inhibitory control

Behaviorally, inhibitory control performance in the flanker task is assessed through response accuracy and reaction times (RT). Participants typically exhibit more errors and longer RTs in the incongruent condition compared with the congruent condition. This pattern indicates that participants must allocate additional attentional and inhibitory resources to respond accurately in the incongruent condition. Consequently, RTs are slower, and error rates are higher due to the cognitive effort required to suppress automatic responses and focus on the target stimulus—an outcome known as the conflict effect.

Event-related potentials (ERPs) recorded via electroencephalography (EEG) are commonly used in conflict resolution tasks to gain deeper insights into the timing and neural mechanisms underlying inhibitory control performance. The two most commonly investigated ERPs in inhibitory control are the N2 and P3b components. The N2 component is a negative potential that occurs 200–350 ms after stimulus onset and is characterized by a frontocentral distribution (16,17). The N2 is an indicator of the level of cognitive conflict. Higher (more negative) N2 amplitude suggests greater conflict detection, increased cognitive control demands, and more effortful inhibition, whereas a decrease may reflect reduced engagement of these processes (18–21). P3b, in turn, is a positive potential occurring 350–600 ms after stimulus onset, with a centroparietal distribution. A larger P3b amplitude indicates the use of more attentional resources during stimulus processing. A larger P3b amplitude is typically observed in incongruent trials than in congruent trials (22). The latency of both components is associated with processing speed, which is usually slower for incongruent trials. An increased latency in N2 suggests slower conflict detection processes (23), whereas increased P3b latency reflects the slower allocation of attentional resources and stimulus classification (24,25).

## Exercise and inhibitory control

PA refers to any bodily movement that increases energy expenditure, including daily activities such as walking. Exercise, a structured and purposeful subset of PA, is performed with the intention of improving physical fitness, which reflects an individual’s physiological capacity, such as cardiovascular fitness. Two principal hypotheses have been proposed to explain the effects of PA or exercise on inhibitory control. The general improvement hypothesis posits that exercise enhances overall cognitive performance, resulting in uniformly faster response times and greater accuracy across various task conditions. In contrast, the selective improvement hypothesis argues that exercise specifically targets certain executive functions—particularly inhibitory control—leading primarily to performance enhancements on more demanding conditions (e.g., incongruent condition). Empirical evidence supports both hypotheses: Engagement in PA or structured exercise has consistently been associated with improved behavioral performance and enhanced neural markers of inhibitory control, with observed benefits encompassing both general cognitive gains and selective improvements specific to inhibition (26).

Specifically, cross-sectional research has linked higher PA levels with enhanced inhibitory control both behaviorally and at the neural level (27–30). Experimental studies have similarly demonstrated positive effects of both acute (23,31,32) and long-term exercise (33,34) on inhibitory control measured with the use of the conflict resolution tasks. In the flanker task, exercise accelerates RTs and improves accuracy in both conditions (congruent and incongruent) (35) or specifically in incongruent condition (20). Enhanced inhibitory control is reflected in ERPs: exercise may decrease N2 amplitude (indicating reduced cognitive conflict) and increase P3b amplitude (suggesting greater attentional resources during the task); the increased cognitive processing speed may also be indicated by reduced latency of both components (23,24).

Although, in some cases, no effects were found (36,37), most research suggests a positive influence of PA levels or exercise on inhibitory control. However, most of the described studies focus on acute exercise interventions rather than long-term exercise training. Among those examining long-term exercise, the focus is typically on elderly individuals or children, and many adopt a cross-sectional design. For instance, a systematic review of 80 randomized controlled trials examining the long-term effects of exercise on cognition in healthy individuals focused almost exclusively on studies involving children, adolescents, and older adults (5). Of 80 included studies, only 2 included young adults; however, neither assessed inhibitory control nor recorded ERPs. On the other hand, as reviewed by Gusatovic et al. (38), some studies involving ERPs and focusing on young adults included clinical populations, such as individuals with substance addiction or depression. As such, the impact of long-term exercise on the inhibitory control of healthy, sedentary individuals is still underexplored, which is surprising given the well-known benefits of chronic PA and increasing rates of sedentary people (4,39).

One of the reasons for the underrepresentation of young adults in research on the cognitive effects of exercise may be that they are generally considered to be at or near their peak cognitive performance (26). However, our study specifically focuses on sedentary young adults, whose lifestyle choices may constrain their cognitive potential, including inhibitory control. Emerging evidence suggests that the cognitive benefits of exercise, particularly improvements in inhibitory control, may be driven at least in part by enhancements in cardiorespiratory fitness (VO_2_max) (40–42). Higher VO_2_max has been associated with increased cerebral blood flow, which supports executive function by optimizing oxygen delivery (43). Enhanced oxygen availability may, in turn, facilitate greater neuronal energy supply and synaptic efficiency, particularly in the prefrontal cortex, a region crucial for inhibitory control (44).

Improved cardiorespiratory fitness has also been linked to higher levels of neurotrophic factors, such as brain-derived neurotrophic factor (BDNF), which supports neuroplasticity, synaptic remodeling, and long-term cognitive adaptation (45). In addition, improvements in cardiorespiratory fitness through aerobic training may contribute to optimized prefrontal dopamine signaling and thus improved performance in tasks requiring cognitive control (45). As a result, higher VO_2_max might also be reflected in modulations of N2 amplitudes, likely driven by strengthened functioning of the prefrontal cortex, as well as in modulations of P3 amplitudes resulting from improved efficiency within the frontoparietal network. Exercise intensity may further shape these effects, as higher-intensity exercise elicits greater BDNF release (46) and more effectively stimulates VO_2_max (47). Thus, sedentary young adults, who typically have lower VO_2_max levels, may possess greater potential for cognitive improvement, as enhancing their cardiorespiratory fitness could lead to improvements in inhibitory control.

## Current study

Given the limited evidence on the effects of long-term exercise on cognition in healthy sedentary adults, we conducted a randomized controlled trial to examine its impact on inhibitory control in this population. Because higher cardiorespiratory fitness is associated with enhanced cognitive performance at both behavioral and neural levels, our intervention aimed to improve fitness through a structured exercise program. Although higher-intensity interval training effectively enhances VO_2_max (47), sedentary individuals typically require a gradual adaptation period to safely and sustainably engage in higher-intensity exercise. Therefore, we implemented a 12-wk intervention comprising moderate-intensity continuous exercise (MCE) during the initial 6 wk to promote preliminary cardiovascular and respiratory adaptations, followed by moderate- to high-intensity interval exercise (MHIE) during the subsequent 6 wk to optimize fitness gains. Inhibitory control was measured before, in the middle (after 6 wk of MCE), and at the end (after 6 wk of MHIE) of the intervention using behavioral responses (RTs and accuracy) and brain activity (amplitude and latency of N2 and P3b components).

We hypothesized that exercise intervention would positively impact inhibitory control, with participants’ performance in the flanker task improving at the midtest (after 6 wk of MCE) compared with baseline and further improving at the posttest (after an additional 6 wk of MHIE) compared with both the midtest and baseline. We expected these improvements to be reflected in one or more of the following outcomes: decreased RT, increased accuracy, reduced N2 amplitude, increased P3b amplitude, and decreased latency of N2 and/or P3b. As such, the design considers multiple patterns of cognitive improvement, distinguishing between inhibition-specific and broader cognitive enhancements. Selective improvements limited to incongruent trials would suggest enhanced inhibitory control, whereas improvements across both congruent and incongruent conditions would reflect general enhancements in cognitive processes. The intervention will be considered effective if RT, accuracy, or both measures show improvement, provided there is no performance trade-off (e.g., improved RT at the expense of decreased accuracy or vice versa). ERP modulations—specifically the N2 (conflict resolution) and P3 (attentional allocation)—will primarily serve to inform behavioral interpretations.

A key innovation of this study is our focus on sedentary and healthy adult participants. Moreover, for the first time during a long-term exercise intervention, we employed the flanker task with ERP recordings in this group. The importance of our research is that exploring the impact of long-term exercise on inhibitory control in sedentary adults may offer a valuable approach to enhancing cognitive health, supporting sustained focus during work, and mitigating the risk of cognitive decline in this vulnerable population.

## METHODS

### Participants

Participants were recruited by the main investigator through advertisements posted on local online platforms and the social media channels of nearby universities between January 2023 and February 2024. The inclusion criteria were as follows: ages 18 to 32 yr, physically inactive (with an approximate PA level <1.4), nonsmokers, no history of claustrophobia, normal or corrected-to-normal vision, no history of neurological disorders, and overall good health. Interested individuals who met these criteria were contacted via email and invited to complete an online prescreening questionnaire. This survey gathered basic demographic information (gender, age, education level, height, and weight), assessed readiness for PA using the Physical Activity Readiness Questionnaire (48), and evaluated PA levels through the Seven-Day Physical Activity Recall Questionnaire (49). Additional questions were included to verify compliance with the inclusion criteria.

Eligible participants were invited to an online meeting to discuss the details of the project with the main investigator. Those who expressed a willingness to participate were subsequently scheduled for a medical examination, which included an exercise electrocardiogram assessment to ensure their safety during the study. After qualifying, participants received a brief introduction to the study and provided informed written consent. The study was conducted following the principles of the Declaration of Helsinki and received approval from the Ethics Committee of the Jagiellonian University in Krakow, Poland.

Following the medical examination, eligible participants were randomized into either the experimental or control group by the main investigator. Although examiners were not blinded to group allocation, standardized protocols were strictly followed to minimize potential bias in data collection. The randomization was initially performed using a simple coin toss (a computer algorithm). However, as the study progressed, a higher dropout rate was observed in the experimental group, resulting in an imbalance in group sizes. To address this issue, we modified the randomization procedure for the final 30 participants, assigning them using a method akin to a coin toss but with three possible outcomes (exercise, exercise, control) to correct the imbalance and ensure an adequate sample size for statistical analysis. An attrition analysis comparing the demographic characteristics of dropped-out participants (*n* = 14) with 1000 bootstrap samples of 14 participants who completed the intervention showed no significant differences in the variables listed in Table 1 (all *P* values >0.05).

**TABLE 1:** Participants baseline characteristics.

	Experimental *M* ± SD	Control *M* ± SD	*N* Exp(♀); Ctrl(♀)	*P*	Total *M* ± SD
Age (yr)	23.50 ± 4.17	23.07 ± 2.45	32(24); 30(20)	0.617	23.29 ± 3.42
Body mass (kg)	69.06 ± 18.27	65.00 ± 10.73	32(24); 30(20)	0.287	67.10 ± 15.12
Height (m)	1.70 ± 0.09	1.70 ± 0.08	32(24); 30(20)	0.887	1.70 ± 0.08
BMI (kg·m^−2^)	23.69 ± 4.83	22.34 ± 2.85	32(24); 30(20)	0.182	23.04 ± 4.02
IQ	13.61 ± 2.49	12.73 ± 3.39	31(23); 30(20)	0.255	13.18 ± 2.97
VO_2_max (mL·kg^−1^·min^−1^)	31.75 ± 5.71	32.28 ± 6.95	32(24); 30(20)	0.748	32.01 ± 6.29
RT, CON (s)	0.37 ± 0.03	0.39 ± 0.05	30(24); 30(20)	0.186	0.38 ± 0.04
RT, INC (s)	0.44 ± 0.03	0.45 ± 0.06	30(24); 30(20)	0.490	0.45 ± 0.05
Accuracy, CON (%)	98.26 ± 3.59	98.68 ± 1.90	30(24); 30(20)	0.576	98.47 ± 2.86
Accuracy, INC (%)	85.74 ± 11.21	89.01 ± 10.07	30(24); 30(20)	0.240	87.37 ± 10.69
P3b LAT, CON (ms)	391.91 ± 39.59	384.95 ± 39.59	29(23); 29(19)	0.466	388.43 ± 39.59
P3b LAT, INC (ms)	429.40 ± 33.36	422.35 ± 27.62	29(23); 29(19)	0.384	425.88 ± 30.49
P3b AMP, CON (μV)	7.72 ± 4.94	7.80 ± 3.41	29(23); 29(19)	0.938	7.76 ± 4.18
P3b AMP, INC (μV)	10.36 ± 4.81	11.00 ± 4.57	29(23); 29(19)	0.606	10.68 ± 4.69
N2 LAT, CON (ms)	322.04 ± 28.65	313.78 ± 45.28	29(23); 29(19)	0.410	317.91 ± 36.97
N2 LAT, INC (ms)	315.46 ± 21.96	314.27 ± 30.40	29(23); 29(19)	0.865	314.87 ± 26.18
N2 AMP, CON (μV)	1.59 ± 3.38	2.10 ± 3.35	29(23); 29(19)	0.565	1.85 ± 3.37
N2 AMP, INC (μV)	−1.21 ± 3.85	−0.13 ± 4.71	29(23); 29(19)	0.345	−0.67 ± 4.28

Baseline cognitive outcomes from the Flanker task measured in the pretest (CON: congruent condition, INC: incongruent condition): RT, Accuracy, P3b LAT (latency of P3b), P3b AMP (amplitude of P3b), N2 LAT (latency of N2), N2 AMP (amplitude of N2). The number of complete datasets for each task is indicated in the last column (Exp: experimental group; Ctrl: control group; the number of females is shown in brackets). The *P* values were obtained using a two-tailed *t*-test for independent means to compare the baseline characteristics between the experimental and control groups.

BMI, body mass index; IQ, Modified Raven’s Matrices test of intelligence (50).

The reported cognitive outcomes were secondary measures in the broader project, for which the sample size was determined based on *a priori* power calculations for the primary outcome. Nevertheless, the study had 75% power to detect an effect of *g* = 0.231, as reported in a meta-analysis on exercise and cognition (5).

Participant characteristics are summarized in Table 1.

**FIGURE 1:**
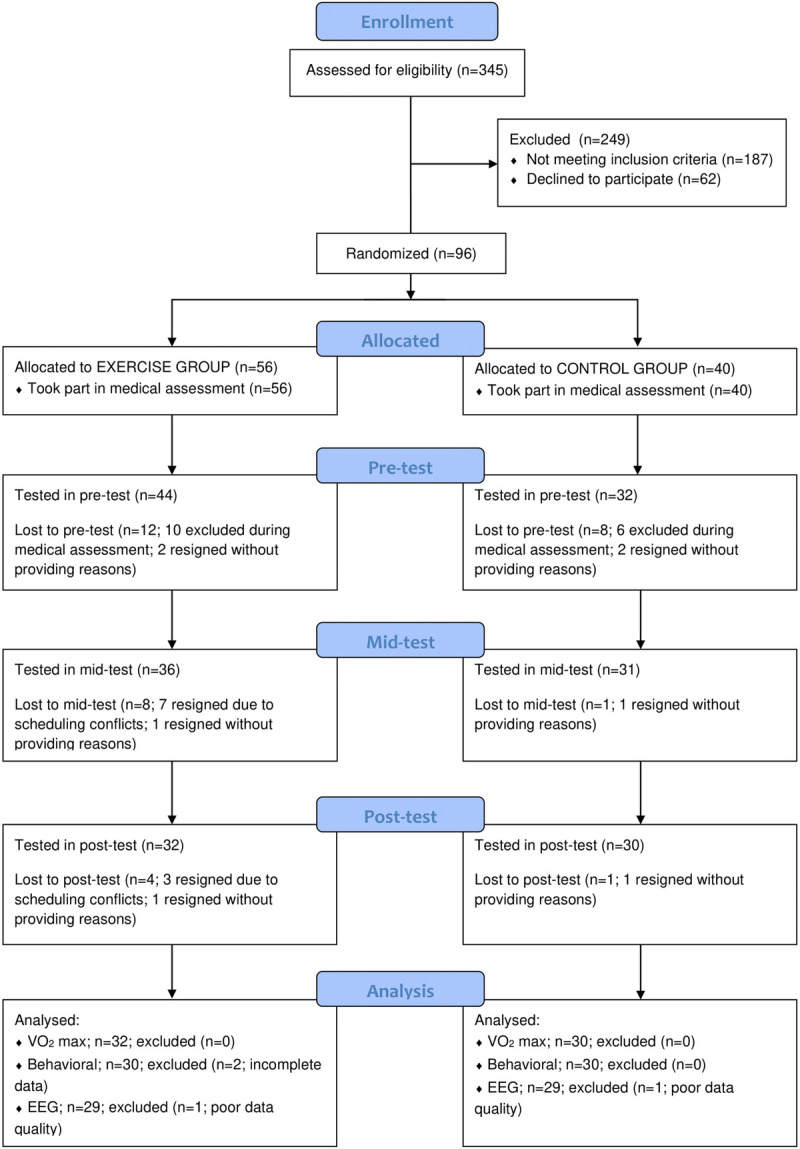
Diagram of participants recruitment.

An overview of the recruitment process and experimental design is presented in Figure 1.

### Experimental design

Participants in both the exercise and control groups attended three testing sessions: before the intervention (pretest), midway through the intervention (midtest, after 6 wk), and following the completion of the intervention (posttest, after 12 wk). During these sessions, outcome measures were collected. Performance diagnostics followed each testing session to assess participants’ cardiovascular fitness levels. These diagnostics were also used for the experimental group to determine appropriate exercise workloads during the intervention. Participants were instructed to refrain from PA for 24 h before each testing session to avoid the acute effects of exercise on the measured outcomes. To minimize the influence of circadian fluctuations on physiological parameters, each testing session for a given participant was scheduled at approximately the same time of day. The trial was not preregistered.

The three testing sessions (pretest, midtest, and posttest) were conducted at the Institute of Psychology at Jagiellonian University in Krakow, Poland. Training sessions for the experimental group were conducted at the Department of Physiology and Biochemistry at the University of Physical Education in Krakow, Poland. The testing period lasted from January 2023 to June 2024.

**FIGURE 2:**
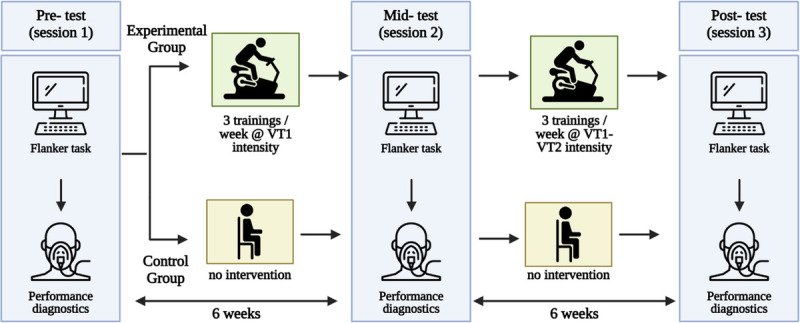
Study intervention overview. Participants were randomly assigned to either the experimental (active) group or the control (passive) group. All participants completed three testing sessions over a 12-wk period: at the start (pretest), after 6 wk (midtest), and at the end of the intervention (posttest). Each testing session included a flanker task and a fitness diagnostic to assess performance and determine the exercise workload for the experimental group. In the experimental group, participants engaged in three stationary cycling sessions per week. During the first 6 wk, each session lasted 30–60 min at a continuous, moderate intensity aligned with the first ventilatory threshold (VT1, 62.7% ± 5.0% HRmax). In the following 6 wk, sessions lasted 60 min, starting with a 6-min warm-up at VT1 power. This was followed by six intervals of 6 min at the second ventilatory threshold (VT2, 81.8% ± 7.48% HRmax) power, with 3-min active recovery periods at VT1 (63.8% ± 7.76% HRmax) power between intervals. The control group (REST) was instructed to maintain their usual habits.

A schematic overview of the study design is provided in Figure 2.

### Cardiovascular fitness diagnostic (VO_2_max and ventilatory thresholds determination)

The maximal oxygen uptake (VO_2_max) was measured using a direct incremental exercise test to volitional exhaustion, designed to assess participants’ aerobic capacity and determine appropriate training loads corresponding to their ventilatory thresholds. The exercise test was conducted on an eBike Comfort bicycle ergometer (GE HealthCare, Chicago, IL). During the test, respiratory and cardiovascular parameters were recorded using a breath-by-breath method via an ergospirometer (MetaLyzer 3R, Cortex, Leipzig, Germany). The parameters measured included minute oxygen uptake (VO_2_), minute carbon dioxide production (VCO_2_), pulmonary ventilation (VE), the fraction of oxygen and carbon dioxide in the exhaled air (FEO_2_% and FECO_2_%), respiratory equivalents for oxygen (VE/VO_2_) and carbon dioxide (VE/VCO_2_), respiratory exchange ratio (RER), and heart rate (HR).

The test protocol began with a 4-min warm-up at 50 W for male participants and 40 W for female participants. Following the warm-up, the workload was systematically increased every 2 min by 25 W for men and 20 W for women, continuing until the participant reached volitional exhaustion. Key metrics determined from the test data included VO_2_max, maximal HR (HRmax), maximal power output (Pmax), the first (VT1) and second ventilatory thresholds (VT2), and the power output at the ventilatory thresholds. To confirm VO_2_max, the following criteria were applied: an RER greater than 1.1, a plateau in oxygen uptake (stability in VO_2_ despite increasing workload), and an HR within 10 bpm of the age-predicted maximum. If no VO_2_ plateau was observed but the other criteria were met, the highest VO_2_ value recorded (VO_2peak_) was accepted as VO_2_max (51). The ventilatory thresholds were identified using the method of respiratory equivalents (52). VT1 was determined at the power output, where the VE/VO_2_ ratio and FEO_2_ reached their minimum values. VT2 was identified at the power output, where the VE/VCO_2_ ratio reached its minimum, and FECO_2_ reached its maximum. During this session, body mass was measured using bioelectrical impedance (IOI-353; Jawon Medical, Kyungsan, Korea), and body height was measured with a stadiometer (Seca 217, Hamburg, Germany) with an accuracy of 0.1 cm.

### Flanker task

The Eriksen Flanker task was presented using PsychoPy v2022.2.4 on a 25-inch Dell Alienware screen positioned approximately 75 cm from the participants. All stimuli were displayed in white font on a gray background. We used a modified version of the flanker task, adapted from Ligeza et al. (20), in which the presentation of the target was delayed to increase task difficulty. This modification enhanced the conflict effect, making inhibiting responses to the flankers more challenging. At the beginning of each trial, a fixation cross appeared at the center of the screen for a random duration between 650 and 1350 ms. Next, four peripheral arrows (flankers) were presented for 500 ms. The target appeared 200 ms after the onset of the flankers and remained on screen for 300 ms until the flankers disappeared. The flankers always pointed in the same direction, either left or right. The target arrow could also point left or right, matching the direction of the flankers in half of the trials (congruent condition) and differing from it in the other half (incongruent condition). The flankers and target always appeared in the same line; however, in half of the trials, they displayed 1/10 of the screen size above the fixation cross and 1/10 of the screen size below in the other half.

After the disappearance of the target and the flankers, the fixation cross appeared on the screen for an additional 1350 ms. If participants did not respond to the target, the trial ended, and another trial began. The response window lasted for 1650 ms, comprising 300 ms during the presentation of both the target and the flankers, followed by 1350 ms during the presentation of the second fixation cross. A response during this period would terminate the trial and initiate the next one.

The task began with instructions in which participants were asked to react as quickly as possible to the targets. Following this, participants completed a short training session consisting of 30 trials. During the training, they received feedback indicating whether their responses were correct, incorrect, or too late. The main task comprised 288 trials divided into three blocks of 96 trials each. After each block, there was a short break, which participants could skip. The entire task lasted about 10 min.

### Intervention

The 12-wk exercise intervention was divided into two 6-wk stages, each with a distinct focus and training intensity (for an intervention overview, see Fig. 2). Participants completed three workouts per week during both stages. Training loads remained consistent throughout each stage and were individually tailored based on the results of performance diagnostics conducted before the start of the stage. Workouts were conducted in groups of 4–8 participants on bicycle ergometers (Wattbike, West Bridgford, UK) in an air-conditioned room with a controlled temperature of 21°C ± 0.5°C and humidity of 40% ± 1%. Each session was supervised by an exercise physiologist. The first stage served as an introductory period, during which participants performed moderate-intensity continuous aerobic exercise (MCE) at a submaximal level, specifically at power corresponding to VT1 (62.7% ± 5.0% HRmax). The duration of each workout gradually increased from 30 min in the first 2 wk to 45 min in the next 2 wk and finally to 60 min in the last 2 wk. The second stage focused on improving aerobic capacity and endurance through MHIE. Participants completed 60-min sessions, beginning with a 6-min warm-up at VT1 power, followed by six intervals of 6 min at VT2 power (81.8% ± 7.48% HRmax), interspersed with 3 min of active recovery at VT1 power (63.8% ± 7.76% HRmax).

The control group was instructed to maintain their usual daily habits throughout the study. Participants in the control group were offered the exercise intervention after completing their passive involvement as part of a waiting list. PA levels in both the control and experimental groups were monitored using activity trackers (Polar, Kempele, Finland). The research team monitored whether participants indeed followed the instructions regarding additional physical activities in their daily lives by inspecting data from the activity trackers weekly. Any deviations from the study protocol were intended to be identified, allowing for early correction if necessary. However, no corrections were required. All participants in the control group adhered to the protocol as instructed. Moreover, all participants in the experimental group completed the required number of training sessions (36 sessions) and did not engage in additional intensive physical activities.

There was a 1-wk break between the two stages, during which a second performance diagnostic was conducted.

### Procedure

At the beginning of each testing session, participants were seated in a softly lit, sound-attenuated, air-conditioned chamber. EEG was recorded using the Biosemi Active Two system. After the equipment was set up, participants completed forms (∼15 min), followed by two tasks (∼10 min) unrelated to the study’s focus. They then performed the Flanker task (∼10 min), followed by three additional tasks, which, like the earlier ones, were beyond the scope of this article and thus not considered. Each session lasted approximately 2.5 h, with the task order of tasks fixed across participants and sessions.

### Electrophysiological recordings and preprocessing

EEG signal was recorded at a sampling rate of 256 Hz from 64 Ag/AgCl scalp electrodes positioned according to the 10–20 system, using the Biosemi Active Two system. Hardware low-pass filtering was applied at 410 Hz using a fifth-order sinc response filter. Horizontal and vertical electrooculograms were recorded bipolarly, with electrodes placed above and below the right eye and at the outer canthi of both eyes

The EEG data were processed offline using custom MATLAB scripts (vR2023b; MathWorks Inc.) in conjunction with the EEGLAB toolbox (v2024.0) and the ERPLAB toolbox (v10.1). The signal was filtered with zero-phase 0.1 Hz high-pass and 30 Hz low-pass filters, then re-referenced to averaged mastoid electrodes. Noisy channels were identified via the clean_rawdata plugin using default parameters and interpolated using spherical interpolation. Epochs were extracted for correct trials, spanning from 200 ms pre-stimulus to 800 ms post-stimulus, with baseline correction applied to the 200-ms pre-stimulus window. After removing epochs affected by amplifier saturation and applying ocular correction using a regression method, any remaining epochs with peak-to-peak voltage fluctuations exceeding 100 μV were discarded. Two participants were excluded because less than 50% of the trials were retained. The average number of analyzed epochs (out of 288) was 258 (standard deviation (SD) = 22, range: 194–288) and did not differ between the experimental and control groups at pretest (259 ± 20 vs 265 ± 19; *P* = 0.21), midtest (257 ± 24 vs 260 ± 22; *P* = 0.61), or posttest (257 ± 24 vs 254 ± 27; *P* = 0.61).

Stimulus-locked ERP averages were computed for each participant, experimental session, and condition (congruent/incongruent) to evaluate N2 and P3b components. N2 was quantified based on its peak latency and mean amplitude within a 25-ms window surrounding the largest ongoing negative peak, identified within a fixed 200- to 350-ms post-stimulus latency window (peak latency: 310 ms; mean window: 285–335 ms). P3b was quantified using a similar approach, identifying the largest ongoing positive peak within a fixed 300- to 600-ms post-stimulus latency window, with mean amplitude calculated within a 50-ms window centered around the peak (peak latency: 400 ms; mean window: 350–450 ms). To assess N2 and P3b, electrode clusters were created by averaging signals within two clusters of electrodes: a frontal cluster (Fz, F1, F2, FCz, FC1, FC2) for N2 and a parietal cluster (Pz, P1, P2, CPz, CP1, CP2) for P3b.

### Data analysis

All statistical analyses and visualizations were conducted using R version 4.0.3 (R Core Team, 2021) and JASP version 0.18.3 (JASP Team, 2022). Outcome variables included VO_2_max (a manipulation check for physical fitness), behavioral outcomes from the flanker task (accuracy and RT), and ERP outcomes (mean amplitude and latency of N2 and P3b components). During data preprocessing, we excluded trials with RT that had at least 3 SDs above or below the mean for each condition of each participant. The normality of all outcome variables was assessed using the Kolmogorov–Smirnov test, with no significant deviations detected (all *P* values > 0.05).

To assess the effects of the intervention on VO_2_max, a 2 × 3 ANOVA was conducted with a two-level between-subjects factor GROUP (experimental, control) and a three-level within-subjects factor SESSION (pretest, midtest, posttest). For the behavioral and ERP outcomes of the flanker task, separate 2 × 3 × 2 ANOVAs were performed, incorporating an additional two-level within-subjects factor CONGRUENCY (congruent, incongruent). The analyses focused primarily on the GROUP–SESSION and GROUP–SESSION–CONGRUENCY interactions, which evaluated the differential impact of the intervention over time between the experimental and control groups. In addition, the main effect of CONGRUENCY was reported. Significant interaction effects were followed up with planned pairwise comparisons (examining differences between pretest, midtest, and posttest across both group and congruency conditions). To minimize the risk of type I errors, we corrected for repeated measures over time using the Benjamini–Hochberg procedure (53) with a threshold of 0.05, applied to a family of 12 comparisons (covering within-group changes over time in both groups).

To assess the robustness of our findings and account for potential bias due to missing data, we conducted a sensitivity analysis that included participants who did not complete the intervention. Missing data were imputed under a conservative assumption that participants who dropped out would have followed trajectories similar to those observed in the passive group. The sensitivity analysis followed the same statistical approach as the primary analysis and is detailed in Supplemental Digital Content (http://links.lww.com/MSS/D243).

## RESULTS

Table 2 presents a summary of the behavioral and EEG results, as well as the VO_2_max outcome.

**TABLE 2:** Means and SD of RT, accuracy, ERP amplitude, and ERP latency in each session.

Session/Condition → Measure/Group ↓	Pretest *M* (SD)		Midtest *M* (SD)		Posttest *M* (SD)	
	CON	INC	CON	INC	CON	INC
RT (s)						
Experimental group	0.370 (0.030)	0.444 (0.031)	0.370 (0.035)	0.428 (0.031)	0.360 (0.035)	0.413 (0.031)
Control group	0.385 (0.054)	0.452 (0.060)	0.388 (0.056)	0.446 (0.059)	0.377 (0.051)	0.435 (0.049)
Accuracy (%)						
Experimental group	98.26 (3.59)	85.74 (11.21)	97.62 (2.09)	87.59 (9.98)	97.20 (2.88)	87.04 (9.17)
Control group	98.68 (1.90)	89.00 (10.07)	97.69 (3.84)	88.03 (11.60)	96.69 (4.84)	84.33 (14.46)
N2 amplitude (μV)						
Experimental group	1.59 (3.38)	−1.21 (3.85)	1.84 (3.55)	−1.25 (4.13)	2.34 (3.19)	1.17 (5.19)
Control group	2.10 (3.35)	−0.13 (4.71)	3.61 (3.83)	−0.02 (4.09)	3.53 (3.57)	−0.01 (4.51)
P3b amplitude (μV)						
Experimental group	7.72 (4.93)	10.36 (4.81)	7.29 (4.26)	10.60 (4.94)	7.48 (4.50)	11.22 (6.07)
Control group	7.80 (3.41)	11.00 (4.57)	8.69 (4.34)	11.53 (4.42)	8.75 (4.09)	12.00 (4.85)
N2 latency (ms)						
Experimental group	322.04 (28.65)	315.46 (21.95)	311.00 (42.75)	313.29 (21.75)	305.25 (45.77)	296.16 (30.88)
Control group	313.78 (45.28)	314.27 (30.40)	303.72 (50.11)	314.16 (21.45)	302.82 (43.69)	307.83 (21.20)
P3b latency (ms)						
Experimental group	391.90 (39.59)	429.40 (33.36)	390.47 (36.24)	424.77 (37.27)	391.68 (37.47)	393.86 (30.56)
Control group	384.95 (32.20)	422.35 (27.62)	378.32 (38.43)	411.28 (32.85)	380.30 (39.69)	417.34 (32.38)

VO_2_max (mL·kg^−1^·min^−1^)	Pretest M (SD)	Midtest M (SD)	Posttest M (SD)
Experimental group	31.75 (5.71)	33.37 (5.42)	36.00 (6.21)
Control group	32.28 (6.95)	31.91 (6.28)	31.41 (5.90)

### VO_2_max

The analysis revealed a significant GROUP–SESSION interaction, *F*(2, 120) = 23.17, *P* < 0.001, *η*^2^ = 0.029. In the experimental group, VO_2_max significantly increased from pretest (*M* = 31.75, SD = 5.71) to midtest (*M* = 33.37, SD = 5.42) (*t* = −3.07, *P* = 0.03) and further increased from midtest to posttest (*M* = 36.00, SD = 6.21) (*t* = −4.99, *P* < 0.001). The difference between pretest and posttest was also significant (*t* = −8.06, *P* < 0.001). In contrast, the control group showed no significant differences between any of the sessions (*M* = 32.28, SD = 6.95 for pretest; *M* = 31.91, SD = 6.28 for midtest; *M* = 31.41, SD = 5.90 for posttest). The GROUP effect was not significant, *F*(1, 60) = 1.54, *P* = 0.22, *η*^2^ = 0.022. However, the SESSION effect was significant, *F*(2, 120) = 10.18, *P* < 0.01, *η*^2^ = 0.013.

### Behavioral Results

**FIGURE 3:**
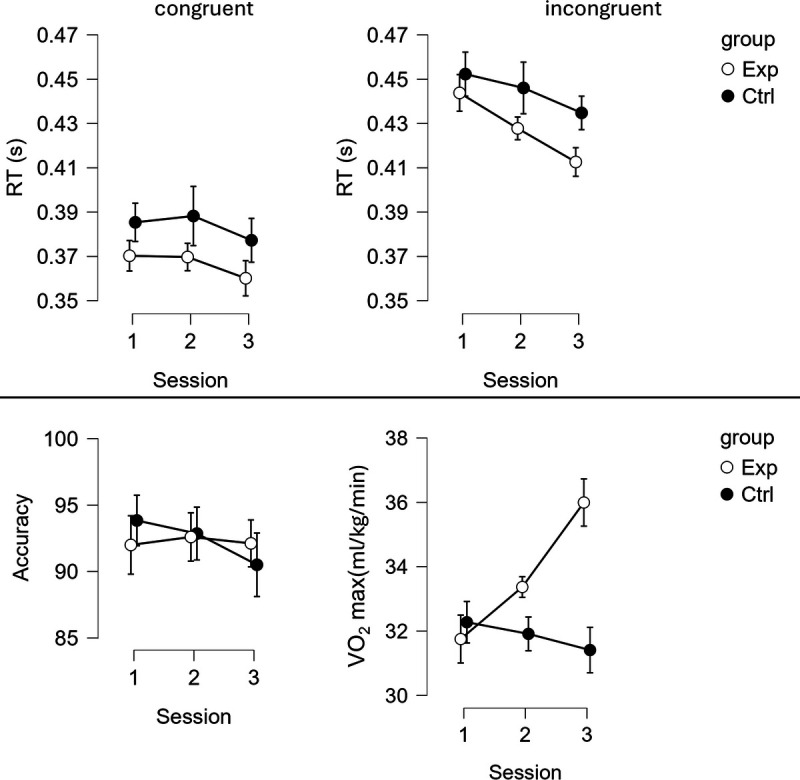
RTs (top) to the target in the congruent (left) and incongruent (right) conditions; accuracy during the task (bottom left) and VO_2_max scores at each testing point (bottom right). White dots represent the experimental group (Exp); black dots represent the control group (Ctrl). Whiskers indicate the 95% confidence intervals.

Figure 3 presents behavioral results (RT and accuracy).

### Reaction Times

The analysis revealed a significant GROUP–SESSION–CONGRUENCY interaction, *F*(2, 116) = 4.227, *P* = 0.017, *η*^2^ = 4.679 × 10^−4^. In the experimental group, RT for incongruent trials decreased from pretest (*M* = 0.444, SD = 0.031) to midtest (*M* = 0.428, SD = 0.031) (*t*(58) = −2.335, *P* = 0.023), from midtest to posttest (*M* = 0.413, SD = 0.031) (*t*(58) = −2.376, *P* = 0.021), and from pretest to posttest (*t*(58) = −5.293, *P* < 0.001). In the control group, RT decreased for incongruent trials between pretest (*M* = 0.452, SD = 0.060) and posttest (*M* = 0.435, SD = 0.049) (*t*(58) = −2.973, *P* = 0.004), but not between pretest and midtest (*M* = 0.446, SD = 0.059) (*t*(58) = −0.913, *P* = 0.365), or between midtest and posttest (*t*(58) = −1.763, *P* = 0.083).

For the congruent condition, no significant differences were found in the experimental group between pretest (*M* = 0.370, SD = 0.030) and midtest (*M* = 0.370, SD = 0.035), midtest and posttest (*M* = 0.360, SD = 0.035), or pretest and posttest (all *P* values > 0.05). Similarly, no significant differences were observed in the control group for the congruent condition between pretest (*M* = 0.385, SD = 0.054) and midtest (M = 0.388, SD = 0.056), midtest and posttest (*M* = 0.377, SD = 0.051), or pretest and posttest (all *P* values > 0.05).

The GROUP–SESSION interaction was not significant, *F*(2, 116) = 0.413, *P* = 0.663, *η*^2^ = 9.833 × 10^−4^. We found a significant main effect of CONGRUENCY, *F*(1, 58) = 541.691, *P* < 0.001, *η*^2^ = 0.309, with RT shorter for congruent trials (*M* = 0.375, SD = 0.039) than incongruent trials (*M* = 0.436, SD = 0.041).

### Accuracy

The analysis revealed no significant GROUP–SESSION–CONGRUENCY interaction, *F*(2, 116) = 2.988, *P* = 0.054, *η*^2^ = 0.003. However, we found a significant GROUP–SESSION interaction, *F*(2, 116) = 5.093, *P* = 0.008, *η*^2^ = 0.005. In the experimental group, accuracy did not differ across all measurement points (pretest: *M* = 92.002, SD = 6.440; midtest: *M* = 92.603, SD = 5.573; posttest: *M* = 92.120, SD = 5.452; all *P* values > 0.05). In the control group, accuracy decreased between midtest (*M* = 92.859, SD = 6.644) and posttest (*M* = 90.509, SD = 8.651) (*t*(58) = −3.742, *P* < 0.001), and between pretest (*M* = 93.843, SD = 5.670) and posttest (*t*(58) = −3.940, *P* < 0.001), but not between pretest and midtest (*t*(58) = −1.222, *P* = 0.227).

We also found a significant main effect of CONGRUENCY, *F*(1, 58) = 82.728, *P* < 0.001, *η*^2^ = 0.299, with accuracy higher in congruent trials (*M* = 97.689, SD = 2.787) than incongruent trials (*M* = 86.956, SD = 10.239).

### EEG Results

**FIGURE 4:**
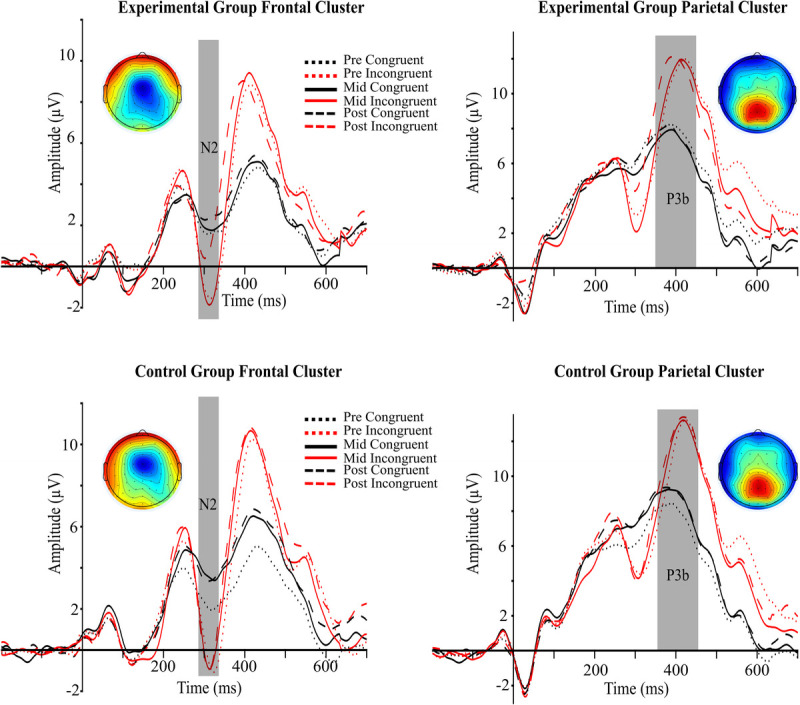
Grand-averaged ERP waveforms. ERP waveforms in response to flanker targets are shown for the experimental group (top row) and control group (bottom row), averaged at frontal (left panels: Fz, F1, F2, FCz, FC1, FC2) and parietal (right panels: CPz, CP1, CP2, Pz, P1, P2) electrode clusters. The N2 (285–335 ms) and P3b (350–450 ms) components are highlighted in gray. Each panel displays waveforms across three testing sessions: pretest (dotted), midtest (solid), and posttest (dashed). Black lines indicate congruent trials; red lines indicate incongruent trials. Scalp maps show the incongruent–congruent difference waves for the N2 and P3b components, averaged across sessions.

Figure 4 presents ERP responses to targets in the Flanker task.

### Amplitude

**FIGURE 5:**
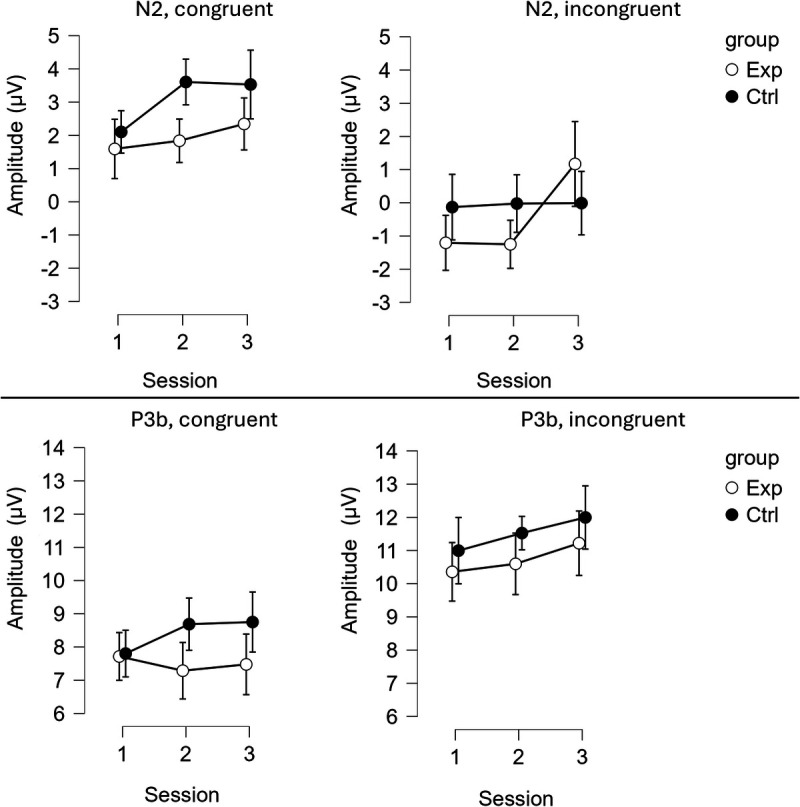
The amplitude of the N2 component (the top section of the graph) and the P3b component (the bottom section of the graph) in response to the target across both conditions (left: congruent; right: incongruent) and the three sessions. White dots represent the experimental group (Exp); black dots represent the control group (Ctrl). Whiskers indicate the 95% confidence intervals.

Figure 5 presents the amplitude of N2 and P3b components in response to the target.

#### N2

The analysis revealed a significant GROUP–SESSION–CONGRUENCY interaction, *F*(2, 112) = 6.92, *P* = 0.001, *η*^2^ = 0.005. In the experimental group, there were no difference in N2 amplitude in response to congruent trials between pretest (*M* = 1.59, SD = 3.38) and posttest (*M* = 2.34, SD = 3.19) (*t*(56) = 1.60, *P* = 0.115), as well as between pretest and midtest (*M* = 1.84, SD = 3.55) (*t*(56) = 0.66, *P* = 0.514) or midtest and posttest (*t*(56) = 1.08, *P* = 0.287).

For incongruent trials, N2 amplitude became more positive from midtest (*M* = −1.25, SD = 4.13) to posttest (*M* = 1.17, SD = 5.19) (*t*(56) = 3.62, *P* < 0.001), and from pretest (*M* = −1.21, SD = 3.85) to posttest (*t*(56) = 3.56, *P* < 0.001), with no significant changes between pretest and midtest (*t*(56) = −0.10, *P* = 0.92).

In the control group, N2 amplitude in response to congruent trials became more positive from pretest (*M* = 2.10, SD = 3.35) to midtest (*M* = 3.61, SD = 3.83) (*t*(56) = 4.03, *P* < 0.001) and from pretest to posttest (*M* = 3.53, SD = 3.57) (*t*(56) = 3.04, *P* = 0.004), with no significant change between midtest and posttest (*t*(56) = −0.16, *P* = 0.87). For incongruent trials, no significant differences were observed across sessions (all *t* < 0.24, all *P* > 0.81).

The GROUP–SESSION interaction was not significant, *F*(2, 112) = 2.81, *P* = 0.065, *η*^2^ = 0.005. The main effect of CONGRUENCY was significant, *F*(1, 56) = 62.09, *P* < 0.001, *η*^2^ = 0.105, with N2 amplitude being more negative for incongruent (*M* = −0.241, SD = 3.988) than for congruent trials (*M* = 2.502, SD = 3.229) (*t*(56) = −7.880, *P* < 0.001).

#### P3b

Neither the GROUP–SESSION–CONGRUENCY interaction nor the GROUP–SESSION interaction was significant (*F*(2, 112) = 1.57, *P* = 0.213, *η*^2^ < 0.001; *F*(2, 112) = 0.854, *P* = 0.428, *η*^2^ = 0.001, respectively). However, the main effect of CONGRUENCY was significant (*F*(1, 56) = 92.81, *P* < 0.001, *η*^2^ = 0.106), with P3 amplitude being more positive for incongruent trials (*M* = 11.115, SD = 4.640) compared with congruent trials (*M* = 7.953, SD = 3.998).

### Latency

**FIGURE 6:**
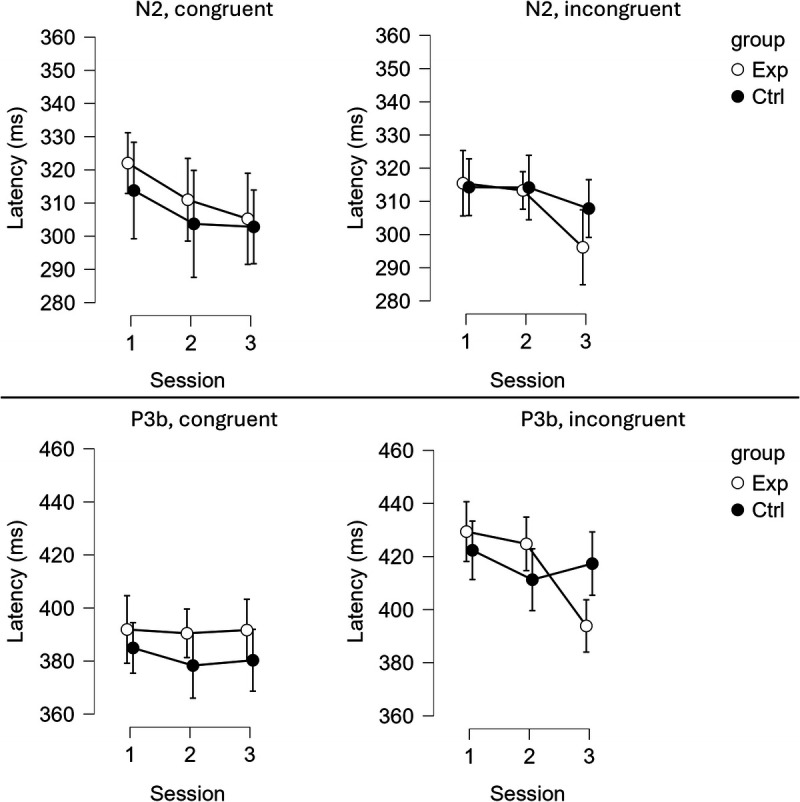
The latency of the N2 component (the top section of the graph) and the P3b component (the bottom section of the graph) across both conditions (left: congruent; right: incongruent) and the three sessions. White dots represent the experimental group (Exp); black dots represent the control group (Ctrl). Whiskers indicate the 95% confidence intervals.

Figure 6 presents the amplitude of N2 and P3b components in response to the target.

#### N2

Neither the GROUP–SESSION–CONGRUENCY interaction nor the GROUP–SESSION interaction reached significance (*F*(2, 112) = 0.190, *P* = 0.827, *η*^2^ < 0.001; *F*(2, 112) = 0.712, *P* = 0.493 *η*^2^ = 0.003, respectively). Similarly, the main effect of CONGRUENCY was not significant, *F*(1, 56) = 0.013, *P* = 0.910, *η*^2^ < 0.001.

#### P3b

The analysis revealed a significant GROUP–SESSION–CONGRUENCY interaction, *F*(2, 112) = 9.041, *P* < 0.001, *η*^2^ = 0.012. In the experimental group, P3 latency in response to congruent trials remained stable across sessions (pretest: *M* = 392, SD = 40; midtest: *M* = 390, SD = 36; posttest: *M* = 392, SD = 37) (all *t* < 0.198, all *P* > 0.84). However, for incongruent trials, P3 latency significantly decreased from midtest (*M* = 425, SD = 37) to posttest (*M* = 394, SD = 31) (*t*(56) = −4.38, *P* < 0.001), and from pretest (*M* = 429, SD = 33) to posttest (*t*(56) = −4.92, *P* < 0.001), with no significant difference between pretest and midtest (*t*(56) = −0.82, *P* = 0.414).

In the control group, P3 latency for congruent trials did not differ significantly across sessions (pretest: *M* = 385, SD = 32; midtest: *M* = 378, SD = 38; posttest: *M* = 380, SD = 40) (all *t* < 0.32, all *P* > 0.38). Similarly, for incongruent trials, no significant changes were observed across sessions (pretest: *M* = 422, SD = 28; midtest: *M* = 411, SD = 33; posttest: *M* = 417, SD = 32) (all *t* < 0.86, all *P* > 0.054).

### Additional Analyses

The sensitivity analysis, which accounted for dropouts, yielded results consistent with the main analysis, confirming the robustness of our findings (see Supplemental Digital Content, http://links.lww.com/MSS/D243).

## DISCUSSION

### General remarks

We examined the impact of a long-term physical exercise intervention on inhibitory control in sedentary, healthy adults randomly assigned to either a 12-wk exercise program or a control group maintaining their usual habits. A novel aspect of this study was the inclusion of this specific population. Our results demonstrate that exercise improved both behavioral and neuroelectrical indices of inhibitory control. Specifically, participants in the exercise group showed reduced RTs on incongruent trials without compromising accuracy, decreased N2 amplitudes, and faster P3b latencies in incongruent trials, suggesting positive influences on inhibitory control. Hereinafter, we discuss these results in greater detail.

### Behavioral findings

The results demonstrated a significant reduction of RT in incongruent trials in the experimental group across all measurement points (pretest to midtest, midtest to posttest, and pretest to posttest), indicating progressive improvements in task performance. In contrast, the control group only showed a significant reduction in RT from pretest to posttest, which may reflect a learning effect and highlight a more pronounced improvement in the experimental group. This improvement is restricted to incongruent trials, which require greater cognitive control due to the need to resolve conflicting information. Thus, the findings suggest that the exercise intervention specifically enhanced participants’ inhibitory control capabilities rather than overall task performance, as could be marked by reduced RT in both congruent and incongruent trials. Alternatively, the lack of RT differences for congruent trials across groups and sessions suggests that participants may have reached a performance ceiling during these trials, which demand fewer cognitive resources. This ceiling effect is likely, given the sample of healthy adults, where intervention-related improvements can be harder to detect (26). To increase the task’s inhibitory demands, we modified the flanker task by presenting the flankers before the target stimulus (20). This likely made the incongruent condition challenging enough to reveal the effects of the exercise while simplifying the congruent condition even further.

Notably, the faster RT to incongruent trials in the experimental group were present without a corresponding decline in accuracy, indicating no speed–accuracy trade-off. In contrast, the control group showed decreased accuracy from midtest to posttest, indicating a potential speed–accuracy trade-off. The control group’s decline in accuracy could reflect fatigue or disengagement over time, whereas the sustained accuracy in the intervention group suggests that the exercise not only reduced RT but also helped maintain participants’ ability to respond accurately, possibly by mitigating factors like fatigue or loss of focus.

Overall, the behavioral findings support the expected benefits of long-term exercise on behavioral measures of inhibitory control, particularly in more demanding task conditions. This aligns with other previous reports; however, to our knowledge, this is the first long-term intervention to demonstrate such effects on an inhibitory task in healthy, sedentary adults (5).

### ERP findings

The effects of the intervention on the ERP changes mostly align with behavioral results. The findings revealed alterations in N2 amplitude and P3b latency that suggest potential neural benefits for the exercise group when responding to trials requiring higher levels of inhibitory control.

Specifically, the experimental group demonstrated decreased N2 amplitude from midtest to posttest during incongruent trials but not during congruent trials, indicating decreased cognitive conflict. In contrast, the passive control group did not exhibit any changes in response to incongruent trials, suggesting a lack of modulation in cognitive conflict processing in response to high-conflict trials. Unexpectedly, the control group decreased N2 amplitude from pretest to midtest and pretest to posttest for congruent trials. This finding is unusual because N2 amplitude in response to congruent trials typically remains stable, providing a baseline for comparison with incongruent trials (18). Given the decreased accuracy observed in the control group, the reduction in N2 amplitude during congruent trials may indicate a decline in cognitive control over time under less demanding conditions, potentially reflecting reduced task engagement.

Overall, the N2 amplitude findings indicate neural benefits associated with the intervention and represent novel evidence for this population. Although previous studies have reported changes in N2 amplitude following long-term training, these findings have often involved specific populations, such as individuals with methamphetamine dependence (54) or depression (55), where inhibitory processes may be impaired. In other designs, such as acute exercise interventions or cross-sectional studies, decreased N2 amplitude is typically associated with greater cognitive performance (24,56,57), although contrasting findings have also been reported (20).

Another finding regarding ERPs concerns the reduction in P3b latency observed in the experimental group, from midtest to posttest and pretest to posttest, with no corresponding change in the control group. This reduction indicates that the exercise intervention accelerated neural processing related to attentional allocation in response to incongruent trials, which is mainly consistent with the behavioral improvements in RT. These results align with previous research showing decreased P3b latency following chronic PA interventions in both older adults (58,59) and children (60,61). The present findings extend this evidence to a broader adult population, indicating that exercise may enhance processing speed and confer cognitive benefits across the lifespan.

No intervention effects were found for N2 latency or P3b amplitude. N2 latency, which reflects the speed of information processing during conflict detection, remained unchanged, indicating that the exercise did not affect the early stages of attentional processing. Although N2 latency is not frequently reported in the literature, we are unaware of any studies demonstrating long-term intervention effects on N2 latency. Some research, however, suggests that exercise may induce a decrease in this measure due to acute aerobic exercise (23).

The intervention did not alter the overall allocation of attentional resources, as indicated by the lack of changes in P3b amplitude. This finding contrasts with previous studies, where P3b amplitude is often modulated in response to chronic PA and is associated with higher cardiorespiratory fitness (62). Increases in P3b amplitude following long-term interventions have been reported, especially in children and older adults (60,61,63–65). The discrepancy between our results and those studies may be attributed to differences in study design, task characteristics, or participant demographics. Most studies do not test healthy, sedentary adults, making it possible that our results are, in fact, typical for this research group (20). It is also possible that the enhanced inhibitory control observed in our study, indicated by reduced cognitive conflict (smaller N2 amplitude), reduced the need for increased attentional resources at later stages of stimulus processing.

Overall, the ERP findings suggest more efficient inhibitory control, as reflected by decreased cognitive conflict (decreased N2 amplitude), as well as faster processing (decreased P3b latency) during trials requiring greater inhibitory effort.

### Selective improvements

The behavioral and neural findings contribute to the ongoing debate regarding the nature of exercise’s effects on executive functions. The “general improvement hypothesis” proposes that exercise enhances overall task performance, such as response times and accuracy, irrespective of specific task demands. Conversely, the “selective improvement hypothesis” posits that exercise primarily benefits specific executive functions, such as inhibition. Although both hypotheses find support in the literature, growing evidence suggests that exercise predominantly enhances general task performance. For instance, a meta-analysis of 80 exercise interventions examining long-term effects on cognitive domains concluded that exercise exerts a general rather than domain-specific effect on cognition (5). However, our results suggest a more nuanced perspective, indicating that exercise may selectively enhance performance under conditions requiring greater inhibitory control, supporting the selective improvement hypothesis.

### Two-stage exercise intervention

An important and novel aspect of this investigation was the two-stage exercise intervention, which comprised 6 wk of MCE followed by 6 wk of MHIE. The rationale was to maximize the cardiovascular fitness of sedentary participants through a two-phase approach: an introductory phase followed by MHIE phase. As anticipated, both phases increased VO_2_max, with the second phase producing greater improvements. The two phases differed mainly in their exercise intensity, which was carefully assessed and individualized for each participant based on their ventilatory thresholds. Behavioral improvements were observed across both phases, indicating that even MCE can yield benefits in sedentary adults with further improvements following the MHIE. However, neuroelectric changes emerged only during the MHIE, suggesting that more demanding or prolonged exercise interventions, or those leading to greater improvements in VO_2_max, may be required to elicit neural adaptations.

Due to the study design, it remains unclear whether the observed benefits are attributable to the cumulative effect of the entire 12-wk intervention or specifically to the inclusion of MHIE. Starting the intervention from MHIE would not have been feasible for sedentary participants, as the MCE phase was intended to progressively prepare them for the more challenging regimen. Thus, the findings suggest that, although 6 wk of MCE exercise can improve behavioral outcomes, adding an MHIE offers additional benefits, enhancing both behavioral and neural markers of cognitive function. As such, these findings further support previous observations that link higher cardiorespiratory fitness with enhanced inhibitory control (41,42,66).

### VO_2_max and its potential contribution to cognitive improvements

Our findings suggest that improvements in cardiorespiratory fitness may have played a key role in the observed changes in inhibitory control and associated neural markers. VO_2_max increased significantly from pretest to midtest, with an even greater increase from midtest to posttest, mirroring the trajectory of behavioral and ERP changes. Notably, some behavioral effects emerged in the first half of the intervention, whereas further behavioral and ERP improvements were observed primarily from midtest to posttest. This pattern aligns with the notion that enhanced oxygen delivery and utilization over time may have facilitated greater neural efficiency.

Cardiorespiratory fitness is known to influence cerebrovascular function, synaptic plasticity, and neural efficiency, all of which contribute to cognitive performance (43,45). Higher VO_2_max has been associated with increased cerebral blood flow, improved metabolic support for neurons, and enhanced efficiency of prefrontal cortical networks, which are critical for inhibitory control. In addition, exercise-induced increases in BDNF and dopaminergic function may have supported adaptive neural changes over the course of the intervention. These mechanisms may explain why ERP components such as N2 amplitude and P3 latency—which reflect conflict monitoring and efficient stimulus evaluation—showed significant improvements only in the later phase of the intervention, when VO_2_max gains were more pronounced.

### Practical implications

Our findings are particularly significant given the increasing prevalence of sedentary behavior and the critical need for effective interventions to mitigate its detrimental effects on cognitive functioning. Based on our findings, long-term exercise interventions may be recommended to improve cognitive functioning, particularly inhibitory control, in sedentary young adults. Inhibitory control is essential for managing distractions, regulating emotions, and resisting impulses—skills that support self-discipline, long-term goal achievement, and effective decision-making under stress. Improvements in inhibitory control may translate into enhanced performance in demanding environments such as learning, work, or driving. Notably, the cycling ergometer protocol is easily scalable, requires minimal supervision, and can be adapted to various settings. This makes it an excellent candidate for inclusion in health promotion campaigns and interventions to reduce sedentary behavior. Furthermore, the study demonstrates that the intervention can have a progressive nature, sustaining and potentially enhancing its positive effects on both behavioral and neural outcomes over time.

Overall, our findings address recent concerns about limited evidence linking exercise to cognitive benefits in healthy populations. By demonstrating that both MCE and MHIE significantly enhance inhibitory control markers, our study provides robust evidence supporting exercise as a means to improve not only inhibition but also cognitive function in healthy individuals.

### Strengths of the study

The advantage of the described study is that it examined a rarely studied group in this context: healthy, sedentary individuals. Most research on the impact of physical exercise on cognitive functioning focuses on clinical patients (54,55), children (67,68), or elderly individuals (33,69), making our research group unique in this area. To our knowledge, the presented results provide the first evidence of the influence of long-term physical exercise on inhibitory control measured with ERPs in this population. Notably, the randomized design and controlled intervention in our study provide robust, high-quality evidence for proving the relationship between exercise and improvement in cognitive function.

Another strength of the study is the precise and robust exercise protocol. As described previously, the training sessions were divided into MCE and MHIE phases, where exercise intensity was carefully controlled using ventilatory thresholds. As a result, we concluded that, even a lighter exercise protocol, MCE improves inhibitory control, whereas MHIE is necessary for neural adaptations. Moreover, the intervention was randomized and controlled. The quality of our results is also highlighted by the correspondence between behavioral and neural measures. In addition to the positive effects observed in behavioral measures (RT, accuracy), we also observed expected effects in N2 amplitude and P3b latency. Although we did not observe changes in P3b amplitude, the explanation for this effect may lie in the specificity of our research group—perhaps for healthy, young individuals, P3b effects are different from those in clinical groups or children, where this component was commonly explored (42).

### Limitations of the study

A key challenge in this study was the higher dropout rate in the experimental group, likely due to the greater time commitment required, which led some participants to withdraw because of scheduling conflicts with work or academic responsibilities. To mitigate this, we adjusted the randomization probability for the final 30 participants to achieve more balanced group sizes. Importantly, our attrition analysis indicated that dropout was random and did not bias the results. Moreover, sensitivity analyses using a conservative imputation approach confirmed that the observed effects were not driven by attrition (see Supplemental Digital Content, http://links.lww.com/MSS/D243). These findings suggest that, despite common adherence challenges in exercise interventions, the observed benefits are likely to persist in real-world settings. However, future studies might benefit from employing adaptive randomization strategies when differential dropout between groups is anticipated. Such designs allow for real-time adjustment of allocation probabilities, helping to maintain balanced groups throughout the trial without compromising methodological rigor.

Another drawback of this study is that the control group was passive. Participants in the control group did not attend training sessions and dedicated less time to the project than the experimental group. On the other hand, the passive control group is ecologically valid, as it accurately reflects the daily routines of sedentary individuals. A further concern is the lack of examiner blinding to group allocation. Although standardized protocols were strictly followed to minimize bias during data collection, the absence of blinding could still introduce expectancy effects.

Finally, a limitation of this study is the exclusive reliance on self-reported PA data during participant screening, which may introduce bias despite the use of well-established and validated questionnaires. However, the mean VO_2_max score of our sample was considerably lower than the general population averages (70) indicating that participants recruited for our study were indeed sedentary.

## CONCLUSIONS

In conclusion, long-term exercise significantly enhances inhibitory control in healthy, sedentary young adults. Both 6 wk of moderate continuous exercise (MCE) and 12 wk of combined MCE and MHIE are associated with behavioral improvements in inhibitory control. The latter intervention is associated with further behavioral and neuroelectric benefits. As such, interventions using both exercise modalities could be recommended to improve cognition in healthy, sedentary adults.

## References

[1] Isath A Koziol KJ Martinez MW, . Exercise and cardiovascular health: a state-of-the-art review. *Prog Cardiovasc Dis*. 2023;79:44–52. 37120119 10.1016/j.pcad.2023.04.008

[2] Stamatakis E Ahmadi MN Friedenreich CM, . Vigorous intermittent lifestyle physical activity and cancer incidence among nonexercising adults: the UK Biobank Accelerometry study. *JAMA Oncol*. 2023;9(9):1255–9. 37498576 10.1001/jamaoncol.2023.1830 PMC10375384

[3] Feter N Ligeza TS Bashir N, . Effects of reducing sedentary behaviour by increasing physical activity, on cognitive function, brain function and structure across the lifespan: a systematic review and meta-analysis. *Br J Sports Med*. 2024;58(21):1295–306. 39197948 10.1136/bjsports-2024-108444

[4] World Health Organization. *Global Action Plan on Physical Activity 2018–2030: More Active People for a Healthier World*. Geneva: World Health Organization; 2018. p. 101 [cited 2024 Dec 17 ] Available from: https://iris.who.int/handle/10665/272722.

[5] Ludyga S Gerber M Pühse U Looser VN Kamijo K. Systematic review and meta-analysis investigating moderators of long-term effects of exercise on cognition in healthy individuals. *Nat Hum Behav*. 2020;4(6):603–12. 32231280 10.1038/s41562-020-0851-8

[6] Diamond A. Executive functions. *Annu Rev Psychol*. 2013;64(1):135–68. 23020641 10.1146/annurev-psych-113011-143750 PMC4084861

[7] Tiego J Testa R Bellgrove MA Pantelis C Whittle S. A hierarchical model of inhibitory control. *Front Psychol*. 2018;9:1339. 30123154 10.3389/fpsyg.2018.01339 PMC6085548

[8] Dong G DeVito EE Du X Cui Z. Impaired inhibitory control in ‘internet addiction disorder’: a functional magnetic resonance imaging study. *Psychiatry Res*. 2012;203(2–3):153–8. 22892351 10.1016/j.pscychresns.2012.02.001 PMC3650485

[9] Gagne JR Liew J Nwadinobi OK. How does the broader construct of self-regulation relate to emotion regulation in young children? *Dev Rev*. 2021;60:100965.

[10] Monachesi B Grecucci A Ahmadi Ghomroudi P Messina I. Comparing reappraisal and acceptance strategies to understand the neural architecture of emotion regulation: a meta-analytic approach. *Front Psychol*. 2023;14:1187092. 37546477 10.3389/fpsyg.2023.1187092 PMC10403290

[11] Romero-López M Pichardo MC Justicia-Arráez A Bembibre-Serrano J. Reducing aggression by developing emotional and inhibitory control. *Int J Environ Res Public Health*. 2021;18(10):5263. 34063369 10.3390/ijerph18105263 PMC8157160

[12] Eriksen BA Eriksen CW. Effects of noise letters upon the identification of a target letter in a nonsearch task. *Percept Psychophys*. 1974;16(1):143–9.

[13] Stroop JR. Studies of interference in serial verbal reactions. *J Exp Psychol*. 1935;18(6):643–62.

[14] Gomez P Ratcliff R Perea M. A model of the go/no-go task. *J Exp Psychol Gen*. 2007;136(3):389–413. 17696690 10.1037/0096-3445.136.3.389 PMC2701630

[15] Simon JR Wolf JD. Choice reaction time as a function of angular stimulus-response correspondence and age. *Ergonomics*. 1963;6(1):99–105.

[16] Falkenstein M Hoormann J Hohnsbein J. ERP components in Go/Nogo tasks and their relation to inhibition. *Acta Psychol (Amst)*. 1999;101(2–3):267–91. 10344188 10.1016/s0001-6918(99)00008-6

[17] Pires L Leitão J Guerrini C Simões MR. Event-related brain potentials in the study of inhibition: cognitive control, source localization and age-related modulations. *Neuropsychol Rev*. 2014;24(4):461–90. 25407470 10.1007/s11065-014-9275-4

[18] Folstein JR Van Petten C. Influence of cognitive control and mismatch on the N2 component of the ERP: a review. *Psychophysiology*. 2008;45(1):152–70. 17850238 10.1111/j.1469-8986.2007.00602.x PMC2365910

[19] Xie L Ren M Cao B Li F. Distinct brain responses to different inhibitions: evidence from a modified flanker task. *Sci Rep*. 2017;7(1):6657. 28751739 10.1038/s41598-017-04907-y PMC5532368

[20] Ligeza TS Maciejczyk M Kałamała P Szygula Z Wyczesany M. Moderate-intensity exercise boosts the N2 neural inhibition marker: a randomized and counterbalanced ERP study with precisely controlled exercise intensity. *Biol Psychol*. 2018;135:170–9. 29665432 10.1016/j.biopsycho.2018.04.003

[21] Wang D Zhou C Chang Y-K. Acute exercise ameliorates craving and inhibitory deficits in methamphetamine: an ERP study. *Physiol Behav*. 2015;147:38–46. 25846839 10.1016/j.physbeh.2015.04.008

[22] Chang Y Alderman BL Chu C Wang C Song T Chen F. Acute exercise has a general facilitative effect on cognitive function: a combined ERP temporal dynamics and BDNF study. *Psychophysiology*. 2017;54(2):289–300. 27861961 10.1111/psyp.12784

[23] Kao S-C Baumgartner N Noh K Wang C-H Schmitt S. Acute effects of intense interval versus aerobic exercise on children’s behavioral and neuroelectric measures of inhibitory control. *J Sci Med Sport*. 2023;26(6):316–21. 37277231 10.1016/j.jsams.2023.05.003

[24] Drollette ES Scudder MR Raine LB, . Acute exercise facilitates brain function and cognition in children who need it most: an ERP study of individual differences in inhibitory control capacity. *Dev Cogn Neurosci*. 2014;7:53–64. 24309300 10.1016/j.dcn.2013.11.001 PMC6987893

[25] Polich J. Updating P300: an integrative theory of P3a and P3b. *Clin Neurophysiol*. 2007;118(10):2128–48. 17573239 10.1016/j.clinph.2007.04.019 PMC2715154

[26] Hillman CH Erickson KI Kramer AF. Be smart, exercise your heart: exercise effects on brain and cognition. *Nat Rev Neurosci*. 2008;9(1):58–65. 18094706 10.1038/nrn2298

[27] Brush CJ Keith LR Santopetro NJ Burani K Hajcak G. Associations between physical activity, sedentary time, and neurocognitive function during adolescence: evidence from accelerometry and the flanker P300. *Prog Brain Res*. 2024;286:151–78. 38876574 10.1016/bs.pbr.2024.01.004

[28] Lennox K Miller RK Martin FH. Habitual exercise affects inhibitory processing in young and middle age men and women. *Int J Psychophysiol*. 2019;146:73–84. 31655182 10.1016/j.ijpsycho.2019.08.014

[29] Van Der Sluys ME Marhe R Van Der Laan PH Popma A Scherder EJA. Brief report: free-living physical activity levels and cognitive control in multi-problem young adults. *Front Hum Neurosci*. 2022;16:994123. 36337855 10.3389/fnhum.2022.994123 PMC9634251

[30] Wunder ML Staines WR. Chronic exercise as a modulator of cognitive control: investigating the electrophysiological indices of performance monitoring. *Front Psychol*. 2022;13:814199. 35450338 10.3389/fpsyg.2022.814199 PMC9016271

[31] Fontes EB Okano AH De Guio F, . Brain activity and perceived exertion during cycling exercise: an fMRI study. *Br J Sports Med*. 2015;49(8):556–60. 23729175 10.1136/bjsports-2012-091924

[32] Griggs MA Parr B Vandegrift NS Jelsone-Swain L. The effect of acute exercise on attentional control and theta power in young adults. *Exp Brain Res*. 2023;241(10):2509–20. 37670008 10.1007/s00221-023-06660-3

[33] Krootnark K Chaikeeree N Saengsirisuwan V Boonsinsukh R. Effects of low-intensity home-based exercise on cognition in older persons with mild cognitive impairment: a direct comparison of aerobic versus resistance exercises using a randomized controlled trial design. *Front Med*. 2024;11:1392429. 10.3389/fmed.2024.1392429 PMC11224483 38975052

[34] You Y Liu J Yao Z Zhang S Chen K Ma X. Neural mechanisms of long-term exercise intervention on cognitive performance among short-sleep young adults: a hemodynamic study. *Sleep Med*. 2023;110:7–16. 37517285 10.1016/j.sleep.2023.07.020

[35] Pontifex MB Saliba BJ Raine LB Picchietti DL Hillman CH. Exercise improves behavioral, neurocognitive, and scholastic performance in children with attention-deficit/hyperactivity disorder. *J Pediatr*. 2013;162(3):543–51. 23084704 10.1016/j.jpeds.2012.08.036 PMC3556380

[36] Gejl AK Bugge A Ernst MT Mortensen EL Gejl KD Andersen LB. Effects of 9 weeks of high- or moderate-intensity training on cardiorespiratory fitness, inhibitory control, and plasma brain-derived neurotrophic factor in Danish adolescents—a randomized controlled trial. *Scand J Med Sci Sports*. 2024;34(8):e14703. 39054765 10.1111/sms.14703

[37] Pensel MC Daamen M Scheef L, . Executive control processes are associated with individual fitness outcomes following regular exercise training: blood lactate profile curves and neuroimaging findings. *Sci Rep*. 2018;8(1):4893. 29559674 10.1038/s41598-018-23308-3 PMC5861091

[38] Gusatovic J Gramkow MH Hasselbalch SG Frederiksen KS. Effects of aerobic exercise on event-related potentials related to cognitive performance: a systematic review. *PeerJ*. 2022;10:e13604. 35846877 10.7717/peerj.13604 PMC9281596

[39] Park JH Moon JH Kim HJ Kong MH Oh YH. Sedentary lifestyle: overview of updated evidence of potential health risks. *Korean J Fam Med*. 2020;41(6):365–73. 33242381 10.4082/kjfm.20.0165 PMC7700832

[40] da Silva LS Neto JBT Junior LPFD Moraes LB Júnior ALMC. Relationship between cardiorespiratory fitness and inhibitory control in children after an acute HIIT session: a cross-randomized trial. *Braz J PhysTher*. 2024;28(Suppl 1):100871.

[41] Tavares VDO Costa KG da Cabral DAR Rego MLM Price M Fontes EB. Cardiorespiratory fitness predicts higher inhibitory control in patients with substance use disorder. *J Clin Sport Psychol* 2020;15(1):4–19.

[42] Raine LB Kao S-C Pindus D, . A large-scale reanalysis of childhood fitness and inhibitory control. *J Cogn Enhanc*. 2018;2(2):170–92.

[43] Salzman T Dupuy O Fraser SA. Effects of cardiorespiratory fitness on cerebral oxygenation in healthy adults: a systematic review. *Front Physiol*. 2022;13:838450. 35309063 10.3389/fphys.2022.838450 PMC8931490

[44] Liu J Min L Liu R, . The effect of exercise on cerebral blood flow and executive function among young adults: a double-blinded randomized controlled trial. *Sci Rep*. 2023;13(1):8269. 37217511 10.1038/s41598-023-33063-9 PMC10203129

[45] McMorris T. Chronic exercise and cognition in humans: a review of the evidence for a neurochemical basis. In: McMorris T (Ed.). *Exercise-cognition interaction: neuroscience perspectives*. Elsevier Academic Press; 2016:167–86. 10.1016/B978-0-12-800778-5.00008-6.

[46] Reycraft JT Islam H Townsend LK Hayward GC Hazell TJ Macpherson REK. Exercise intensity and recovery on circulating brain-derived neurotrophic factor. *Med Sci Sports Exerc*. 2020;52(5):1210–7. 31815833 10.1249/MSS.0000000000002242

[47] Milanović Z Sporiš G Weston M. Effectiveness of high-intensity interval training (HIT) and continuous endurance training for VO_2_max improvements: a systematic review and meta-analysis of controlled trials. *Sports Med*. 2015;45(10):1469–81. 26243014 10.1007/s40279-015-0365-0

[48] Thomas S Reading J Shephard RJ. Revision of the Physical Activity Readiness Questionnaire (PAR-Q). *Can J Sport Sci*. 1992;17(4):338–45. 1330274

[49] Washburn RA Jacobsen DJ Sonko BJ Hill JO Donnelly JE. The validity of the Stanford Seven-Day Physical Activity Recall in young adults. *Med Sci Sports Exerc*. 2003;35(8):1374–80. 12900693 10.1249/01.MSS.0000079081.08476.EA

[50] Marzecová A Bukowski M Correa Á Boros M Lupiáñez J Wodniecka Z. Tracing the bilingual advantage in cognitive control: The role of flexibility in temporal preparation and category switching. *Cogn Psychol*. 2013;25(5):586–604.

[51] Howley ET Bassett DR Welch HG. Criteria for maximal oxygen uptake: review and commentary. *Med Sci Sports Exerc*. 1995;27(9):1292–301. 8531628

[52] Binder RK Wonisch M Corra U, . Methodological approach to the first and second lactate threshold in incremental cardiopulmonary exercise testing. *Eur J Cardiovasc Prev Rehabil*. 2008;15(6):726–34. 19050438 10.1097/HJR.0b013e328304fed4

[53] Benjamini Y Hochberg Y. Controlling the false discovery rate: a practical and powerful approach to multiple testing. *J R Stat Soc Ser B Methodol*. 1995;57(1):289–300.

[54] Wang D Zhu T Zhou C Chang Y-K. Aerobic exercise training ameliorates craving and inhibitory control in methamphetamine dependencies: a randomized controlled trial and event-related potential study. *Psychol Sport Exerc*. 2017;30:82–90.

[55] Olson RL Brush CJ Ehmann PJ Alderman BL. A randomized trial of aerobic exercise on cognitive control in major depression. *Clin Neurophysiol*. 2017;128(6):903–13. 28402866 10.1016/j.clinph.2017.01.023

[56] Pontifex MB Hillman CH. Neuroelectric and behavioral indices of interference control during acute cycling. *Clin Neurophysiol*. 2007;118(3):570–80. 17095295 10.1016/j.clinph.2006.09.029

[57] Stroth S Kubesch S Dieterle K Ruchsow M Heim R Kiefer M. Physical fitness, but not acute exercise modulates event-related potential indices for executive control in healthy adolescents. *Brain Res*. 2009;1269:114–24. 19285042 10.1016/j.brainres.2009.02.073

[58] Cetin E Top EC Sahin G Ozkaya YG Aydin H Toraman F. Effect of vitamin E supplementation with exercise on cognitive functions and total antioxidant capacity in older people. *J Nutr Health Aging*. 2010;14(9):763–9. 21085907 10.1007/s12603-010-0256-x PMC12880339

[59] Chuang L-Y Hung H-Y Huang C-J Chang Y-K Hung T-M. A 3-month intervention of Dance Dance Revolution improves interference control in elderly females: a preliminary investigation. *Exp Brain Res*. 2015;233(4):1181–8. 25595954 10.1007/s00221-015-4196-x

[60] Chang Y-K Tsai Y-J Chen T-T Hung T-M. The impacts of coordinative exercise on executive function in kindergarten children: an ERP study. *Exp Brain Res*. 2013;225(2):187–96. 23239198 10.1007/s00221-012-3360-9

[61] Hillman CH Pontifex MB Castelli DM, . Effects of the FITKids randomized controlled trial on executive control and brain function. *Pediatrics*. 2014;134(4):e1063–71. 25266425 10.1542/peds.2013-3219 PMC4179093

[62] Kao S-C Cadenas-Sanchez C Shigeta TT, . A systematic review of physical activity and cardiorespiratory fitness on P3b. *Psychophysiology*. 2020;57(7):e13425. 31228362 10.1111/psyp.13425

[63] Ludyga S Gerber M Kamijo K Brand S Pühse U. The effects of a school-based exercise program on neurophysiological indices of working memory operations in adolescents. *J Sci Med Sport*. 2018;21(8):833–8. 29358034 10.1016/j.jsams.2018.01.001

[64] Ozkaya GY Aydin H Toraman FN Kizilay F Ozdemir O Cetinkaya V. Effect of strength and endurance training on cognition in older people. *J Sports Sci Med*. 2005;4(3):300–13. 24453535 PMC3887334

[65] Tsai C-L Pan C-Y Chen F-C Tseng Y-T. Open- and closed-skill exercise interventions produce different neurocognitive effects on executive functions in the elderly: a 6-month randomized, controlled trial. *Front Aging Neurosci*. 2017;9:94. 28959200 10.3389/fnagi.2017.00294 PMC5604064

[66] Wengaard EJ Kristoffersen M Harris A Gundersen H. Cardiorespiratory fitness is associated with selective attention and inhibitory control in healthy male high-school students. *Front Hum Neurosci*. 2017;11:330. 28701935 10.3389/fnhum.2017.00330 PMC5487396

[67] Xue Y Yang Y Huang T. Effects of chronic exercise interventions on executive function among children and adolescents: a systematic review with meta-analysis. *Br J Sports Med*. 2019;53(22):1397–404. 30737201 10.1136/bjsports-2018-099825

[68] Zhong X Wang C Xu M Yuan X Jiang C. Physical training improves inhibitory control in children aged 7–12 years: an fNIRS study. *Behav Brain Res*. 2024;463:114902. 38341102 10.1016/j.bbr.2024.114902

[69] Xu L Gu H Cai X, . The effects of exercise for cognitive function in older adults: a systematic review and meta-analysis of randomized controlled trials. *Int J Environ Res Public Health*. 2023;20(2):1088. 36673844 10.3390/ijerph20021088 PMC9858649

[70] Xiang L Deng K Mei Q, . Population and age-based cardiorespiratory fitness level investigation and automatic prediction. *Front Cardiovasc Med*. 2021;8:758589. 35071342 10.3389/fcvm.2021.758589 PMC8767158

